# CDK12‐inactivation‐induced MYC signaling causes dependency on the splicing kinase SRPK1

**DOI:** 10.1002/1878-0261.13666

**Published:** 2024-05-22

**Authors:** Jing Liang, Aishwarya Gondane, Harri M. Itkonen

**Affiliations:** ^1^ Department of Biochemistry and Developmental Biology, Faculty of Medicine University of Helsinki Helsinki Finland

**Keywords:** cyclin‐dependent kinase 12, prostate cancer, SLAM‐seq, splicing, synthetic lethality

## Abstract

Inactivation of cyclin‐dependent kinase 12 (CDK12) characterizes an aggressive sub‐group of castration‐resistant prostate cancer (CRPC). Hyper‐activation of MYC transcription factor is sufficient to confer the CRPC phenotype. Here, we show that loss of *CDK12* promotes MYC activity, which renders the cells dependent on the otherwise non‐essential splicing regulatory kinase SRSF protein kinase 1 (SRPK1). High *MYC* expression is associated with increased levels of SRPK1 in patient samples, and overexpression of MYC sensitizes prostate cancer cells to SRPK1 inhibition using pharmacological and genetic strategies. We show that Endovion (SCO‐101), a compound currently in clinical trials against pancreatic cancer, phenocopies the effects of the well‐characterized SRPK1 inhibitor SRPIN340 on nascent transcription. This is the first study to show that Endovion is an SRPK1 inhibitor. Inhibition of SRPK1 with either of the compounds promotes transcription elongation, and transcriptionally activates the unfolded protein response. In brief, here we discover that *CDK12* inactivation promotes MYC signaling in an SRPK1‐dependent manner, and show that the clinical grade compound Endovion selectively targets the cells with *CDK12* inactivation.

## Graphical abstract

Inactivation of the transcription elongation kinase cyclin‐dependent kinase 12 (CDK12) stimulates MYC signaling. MYC serves as a transcriptional amplifier and makes cancer cells dependent on SRSF protein kinase 1 (SRPK1). We propose that inactivating mutations and deletion of the *CDK12* gene are biomarkers of sensitivity to SRPK1 inhibitors.

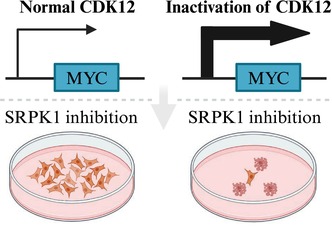

**Abbreviations**
AR‐v7: androgen receptor splice variant v7
CDK12: cyclin‐dependent kinase 12
ChIP‐seq: chromatin immunoprecipitation coupled with massively parallel DNA sequencing
CRPC: castration‐resistant prostate cancer
CSS: charcoal‐stripped FBS
CTG: CellTiter‐Glo
DEGs: differentially expressed genes
DTT: dithiothreitol
ERG: ETS transcription factor ERG
ETV1: ETS variant transcription factor 1
FBS: fetal bovine serum
FC: fold change
FOXA1: Forkhead box A1
GSEA: gene set enrichment analysis
GEO: Gene Expression Omnibus
HOXB13: homeobox protein hox‐B13
MISO: mixture‐of‐isoforms
PC: prostate cancer
RNA Pol II: RNA polymerase II
rMATS: replicate multivariate analysis of transcript splicing
SF3B: splicing factor 3b
SLAM‐DUNK: thiol(SH)‐linked alkylation for the metabolic sequencing of RNA‐Digital Unmasking of Nucleotide conversions in K‐mers
SLAM‐seq: thiol(SH)‐linked alkylation for the metabolic sequencing of RNA
SRPK1: SRSF protein kinase 1
SRPK2: SRSF protein kinase 2
SRPK3: SRSF protein kinase 3
SRSF1: serine‐ and arginine‐rich splicing factor 1
SU2C/PCF: Stand Up to Cancer/Prostate Cancer Foundation
UPR: unfolded protein response
VEGF: vascular endothelial growth factor
4‐sU: 4‐thiouridine

## 1 Introduction

Prostate cancer (PC) is the most common cancer in men, and currently, the therapy‐induced castration‐resistant PC (CRPC) has no curative measures [1, 2]. CRPC cells rewire their transcriptomes to allow continuous proliferation despite the presence of anti‐androgens. One of the hallmark features of PC cells is elevated transcription to maintain high levels of expression of the pro‐proliferative and anti‐apoptotic genes that typically have short half‐lives. Unexpectedly, inactivation of the major transcription elongation kinase, cyclin‐dependent kinase 12 (*CDK12*) is associated with a more aggressive cancer‐phenotype [3, 4]. Inhibition of CDK12 activity promotes transcription of the short genes and simultaneously impairs the expression of the particularly long genes [5, 6]. Increased transcription burdens the spliceosome, and the splicing dysregulation becomes more severe as CRPC progresses [7]. In essence, spliceosome represents a candidate drug target against CRPC.

Spliceosome‐targeting therapies provide an intriguing approach to kill cancer cells. Compounds against the universal component of the spliceosome, SF3B, have shown significant anti‐tumor effects *in vitro* and also in animal models [7]. However, in clinical trials, these compounds have resulted in on‐target undesirable side effects and severe toxicity [8, 9]. In this context, it is of a high interest to seek for splicing‐regulators that would be hyper‐activated in CRPC and ideally be non‐essential for the normal cells.

Serine/Arginine‐Rich Splicing Factor Kinase 1 (SRPK1) is overexpressed in aggressive prostate cancer but the gene is non‐essential in most cell types [10, 11]. SRPK1 and SRPK2 regulate the nuclear import of serine‐ and arginine‐rich proteins [12, 13]. The role of SRPK1 in cancer is better understood, and the kinase is known to hyper‐phosphorylate SRSF1. Inhibition of SRPK1 switches the SRSF1‐mediated splicing of the vascular epithelia growth factor (VEGF) mRNA from the predominant pro‐angiogenic VEGF_165a_ isoform to the anti‐angiogenic VEGF_165b_ isoform [14, 15]. However, no study has systematically evaluated how SRPK1 inhibition affects RNA polymerase II output and the overall transcriptional program; therefore, it is not known what transcription factors are particularly dependent on this kinase.

SRPK1 represents a candidate drug target against CRPC and there are a number of small molecule inhibitors available to decrease its activity. In addition to cancer, SRPK1 inhibitors could be potentially used to control the excessive vascularization observed in diabetic retinopathy [16]. Unfortunately, while the existing SRPK1 inhibitors provide suitable tools for scientific research, none of them has been tested in clinical trials [17]. We searched the clinical trials database (ClinicalTrials.gov) to ask if SRPK1 is used as a biomarker in any. Unexpectedly, we identified a study carried out by Scandion Oncology (NCT04247256), which claims to test the efficacy of an SRPK1 inhibitor SCO‐101 (Endovion) in pancreatic cancer patients. However, no data has ever been disclosed to show that Endovion actually targets SRPK1. This compound was proposed as a candidate therapy against sickle cell anemia and while its development was discontinued some 20 years ago, the safety was confirmed in four separate clinical trials on healthy volunteers [18]. Currently, there is one active Phase 1 and one active Phase 2 clinical trial assessing Endovion against pancreatic cancer and metastatic colorectal cancer, respectively (NCT04652206 and NCT04247256).

In this study, we set out to probe if Endovion is actually an SRPK1 inhibitor and to establish how depletion of the SRPK1 activity affects nascent transcription. We confirm that both Endovion and the well‐validated SRPK1 inhibitor SRPIN340 induce highly similar effects on the nascent transcription. Inhibition of SRPK1 with either of the compounds promotes transcription of the genes with a particularly high number of exons and activates the unfolded protein response. Endovion is combinatorically lethal with CDK12 inhibition, as we have previously reported for the SRPK1 inhibitor SRPIN340. We show that the decrease in CDK12 activity promotes MYC signaling in an SRPK1‐dependent manner and that the high activity of SRPK1 is essential for the proliferation of prostate cancer cells with high MYC expression. Finally, we propose that inactivation of *CDK12* is a biomarker of sensitivity against Endovion.

## 2 Materials and methods

### 2.1 Cell culture and proliferation assays

22RV1 (Research Resource Identifier: CVCL_1045) and C4‐2 (Research Resource Identifier: CVCL_4782) cell lines were obtained from the American Tissue Culture Collection (frozen stocks were prepared from the vial received and not allowed to grow for more than 25 passages, which assures the authentity of the cell lines), and the cells were maintained in RPMI medium supplemented with 10% fetal bovine serum (FBS). LNCaP‐MYC (derived from Research Resource Identifier: CVCL_0395 cell line but does not have its own Research Resource Identifier) cell line has been described by Ramos‐Montoya et al. [19], and these cells were maintained as described by the authors. Mycoplasma testing is performed periodically and/or when reason to suspect (we have not had mycoplasma). For androgen‐starvation experiments, LNCaP‐MYC cells were kept in the charcoal‐stripped FBS (CSS) first for 24 h (transfection) and then for 96 h (compound treatment). Transfection of Ambion® Silencer Select siRNA against SRPK1 (s13450) was achieved using RNAiMax. For viability assays, knockdown was performed for 24 h, after which MYC (2 μG∙μL^−1^ doxycycline) was overexpressed for 4 days. Next, CellTiter‐Glo® 2.0 assay (Promega) was used according to the manufacturer's instructions in technical triplicates in 384‐well plates and the data presented is from at least three biological replicates. For western blot experiments, knockdown was performed for 72 h, after which MYC was overexpressed for 24 h in LNCaP‐MYC cell line. Cell lysates for western blot were prepared as previously described (except no sonication) [20]. Antibodies used were from Proteintech: SRPK1 (14073‐1‐AP), from Cell Signaling technology: MYC (CST5605), from Abcam: Actin (ab49900), and from MERCK: anti‐phosphoepitope SR proteins (MABE50). Densitometry was used to measure the signal intensity (Image Lab; Bio‐Rad). THZ531, SRPIN340, Endovion, and Doxycycline were obtained from MedChemExpress. Crystal violet staining assay was used to measure the colony formation ability of cells as previously described [21].

### 2.2 Preparation of samples for SLAM‐seq

22RV1 cells were grown in 12‐well plates for 1 day. The next day, cells were treated for 4 h with 20 μM SRPIN340, 50 μM Endovion, 100 nM THZ531 or combination of SRPIN340 with THZ531 and Endovion with THZ531. For the last 10 min, cells were treated with 4‐thiouridine (4sU, obtained from ThermoFisher Scientific). RNA was purified using illustraMiniSpin‐kit (GE Healthcare) according to manufacturer's instructions except all the buffers contained 0.1 mM DTT. The purified RNA was treated with 10 mM iodoacetamide as previously described [22]. Subsequently, the alkylated RNA was ethanol precipitated. Library preparation and sequencing were purchased as service, in brief: 300 ng of RNA was subjected to Quant‐seq 3′‐end mRNA library preparation according to manufacturer's instructions (Lexogen, Vienna, Austria, protocol version 015UG009V0251). Dual indexing and UMI modules were used in the library construction process. Library quality check was performed using LabChip GX Touch HT High Sensitivity assay (PerkinElmer, Hopkinton, MA, USA) and libraries were pooled based on the concentrations acquired from the assay. The library pool was quantified using Collibri Library Quantification kit (Thermo Fisher Scientific, Waltham, MA, USA) and then sequenced on the Illumina NovaSeq6000 system using S1 flow cell (Illumina, San Diego, CA, USA) with v1.5 reagents. Read length for the single‐end run was 101 bp.

### 2.3 Bioinformatics

Chemdraw (RRID:SCR_016768) was used to draw Endovion and SRPIN340 structures. For SLAM‐seq analysis, we used raw FASTQ files to call the nascent transcripts with SLAM‐DUNK [23]. DESeq2 [24] calls the differentially labeled transcripts from the count files, which were generated by SLAM‐DUNK [23]. Threshold for *P*‐value of 0.05 and log_2_ fold change of either 1 or −1 was applied to define a transcript as significantly up‐ or downregulated. We analyzed our SLAM‐seq data using DESeq2 [24] to generate traditional RNA‐seq expression profiles (that is, without the SLAM‐DUNK [23] step). Briefly, we aligned the raw FASTQ files to GRCh38 reference using Bowtie2 [25]. The aligned SAM files were converted to their binary versions (BAM) using SAMtools [26]. Corresponding index files and the BAM files were used as input to DESeq2 to call the differentially expressed genes (DEGs). Fgsea [27] was used to perform gene set enrichment analysis (GSEA). Threshold of *P*_adj_‐value <0.05 was selected to define the significantly enriched hallmark pathways. Gene length information of the protein‐coding genes was obtained from [28], and we trichotomized gene lengths to short: <1 kb, medium: 1–15 kb, and long: >15 kb (from that article, Table S1). The exon count for genes was obtained from [29]. The effect of CDK12 inhibitor on the expression levels of the selected gene sets was evaluated using previously reported datasets: MYC targets V1 and androgen response are the genes from GSEA (GSE121474), and for the following we used ChIP‐seq data: AR‐v7 [30], FOXA1 (GSE148926), ERG (GSM3223717), ETV1 (GSM1145322) and HOXB13 (GSE96652). The volcano‐, box‐, violin‐, and scatter‐plots were generated in R (4.2.0).

RNA‐seq data from LNCaP cells treated with 0.5 μM THZ531 for 6 h was downloaded from Genome Sequence Archive (HRA000724). The FASTQ files were aligned using Bowtie2 [25] with default parameters. The aligned SAM files were converted to BAM and later sorted using SAMtools [26]. The sorted BAM files were used to call the differentially expressed genes using DESeq2 [24].

## 3 Results

### 3.1 Endovion inhibits SRPK1

SRPK1 is an attractive drug target because the increased expression of this gene correlates with aggressive CRPC features and the gene is non‐essential to most cells. We searched the clinical trials database (ClinicalTrials.gov) and identified a study (NCT04247256), which claims to evaluate the potential of an SRPK1 inhibitor against pancreatic cancer. This compound is Endovion [18], but no information has been disclosed in the literature to show that it actually targets SRPK1. A search of the patent literature revealed that Scandinavian Oncology has demonstrated Endovion's ability to decrease SRPK1 activity [31].

**Fig. 1:**
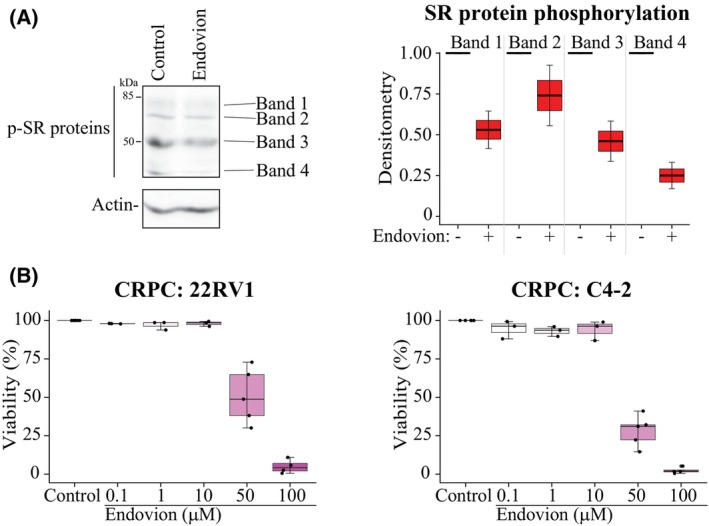
Endovion targets SRSF protein kinase 1 and is toxic to castration‐resistant prostate cancer (CRPC) cells. (A) Endovion decreases phosphorylation of Serine/Arginine‐Rich Splicing Factor (SR) proteins. 22RV1 prostate cancer cells were treated with 25 μM Endovion for 4 h and analyzed using western blotting. Densitometry was used to record signal intensity and the abundance of the phosphorylated SR proteins is presented as percent of control (the signal from both control‐ and Endovion‐treated samples was first normalized to Actin, n: 2). (B) Cell viability of CRPC cells after 4 days of treatment (depending on the dose, either 3 or 5 biological replicates are plotted, each three technical replicates).

First, we set out to confirm that Endovion inhibits SRPK1 in prostate cancer cells and to assess if it has anti‐proliferative effects. SRPK1 phosphorylates serine/arginine‐rich splicing factors (SR proteins), which can be used as a read‐out of its activity. Indeed, 25 μM Endovion decreased the phosphorylation levels of different SR proteins even down to 17% after 4 h of treatment (Fig. 1A). We reasoned that by genetically decreasing the levels of SRPK1, we should further sensitize prostate cancer cells to Endovion, which we confirmed to be the case (Fig. S1). Next, we performed proliferation assays to identify a dose of Endovion that has clear effect on proliferation of the CRPC cells. These experiments revealed that 50 μM Endovion suppresses proliferation of CRPC models by 50–75% (Fig. 1B).

To summarize the major findings so far, Endovion decreases proliferation rate of CRPC cells and is in clinical development against other cancers. Potential additional targets of Endovion are poorly characterized but the compound is also known to inhibit drug efflux pumps [18]. We reasoned that because this compound has been confirmed to be safe in multiple clinical trials, it is not of a high priority to establish what the additional target(s) may be. More important would be to understand how cancer cells respond to this compound, and to identify a biomarker of sensitivity.

### 3.2 SRPK1 promotes transcription elongation of the long genes with a high number of exons

**Fig. 2:**
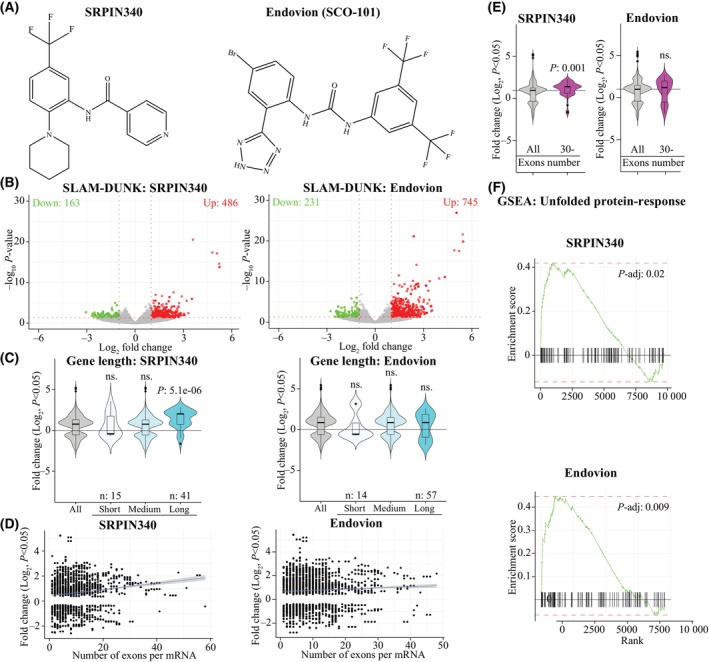
SRSF protein kinase 1 (SRPK1) inhibition stimulates transcription of the particularly long genes with high number of exons. For all of the data in this figure, n: 2. (A) Molecular structures of SRPIN340 and Endovion. (B) Analysis of the nascent transcription (SLAM‐seq) after 4 h treatment with 20 μM SRPIN340 or 50 μM Endovion. The genes decreased or increased significantly (*P* < 0.05) and less/more than log_2_ fold change ±1 are highlighted with green and red colors, respectively. (C) SRPK1 inhibition decreases transcription elongation of the particularly short genes. Genes were grouped into three categories and the significantly affected genes based on SLAM‐seq in each categories are plotted. Significance was assessed using Student's *t*‐test, ns, non‐significant. (D) SRPK1 inhibition stimulates transcription of genes with a high number of exons. Genes whose transcription changes significantly in response to SRSF protein kinase 1 inhibition are correlated with the exon counts. (E) SRPK1 inhibition stimulates transcription of genes with more than 30 exons (data obtained from 2D). Significance was assessed using Student's *t*‐test. (F) Gene set enrichment analysis of the SLAM‐seq data identifies unfolded protein response (UPR) as the most significantly affected gene set in response to both SRPIN340 and Endovion treatments. All data presented in this figure are generated using 22RV1 cell line. Statistical test used: Kolmogorov–Smirnov test.

We used metabolic labeling of transcription, SLAM‐seq, to probe if Endovion causes similar transcriptional effects as does the well‐validated SRPK1 inhibitor SRPIN340 [32]. Structurally, the two compounds are clearly distinct (Fig. 2A). In SLAM‐seq, the nascent RNA is labeled prior to RNA purification, which enables subsequent computational discovery of the newly made mRNAs [22]. Interestingly, most of the mRNAs were upregulated in response to both Endovion and SRPIN340 treatments (Endovion: 745 up and 231 down; SRPIN340: 486 up and 163 down, log_2_FC ± 1, *P* < 0.05, Fig. 2B). This was unexpected, because splicing is vital for generating the productive mRNAs and both Endovion and SRPIN340 decrease proliferation of the CRPC cells.

We hypothesized that SRPK1 inhibition affects transcription in a gene length‐dependent manner because long genes are more complex to process and would therefore require high activity of the spliceosome. To test if SRPK1 inhibition affects transcription in a gene length‐dependent manner, we trichotomized genes into short, medium and long. Interestingly, both Endovion and SRPIN340 inhibited transcription of the short mRNAs (Fig. 2C). In addition, particularly SRPIN340 significantly stimulated transcription of the long genes. This is the first time that inhibition of splicing has been shown to cause gene length‐dependent effects, and indicates that splicing stalls the speed of RNA Pol II.

If transcription is decoupled from splicing this should be highly detrimental to cells due to accumulation of the wrong open reading‐frames and premature stop‐codons. We reasoned that the genes stimulated in response to SRPK1 inhibition should be largely intronless, because SRPK1 is a splicing regulatory kinase. To test this, we evaluated nascent RNA synthesis with respect to exon number. In contrast to our expectation, both Endovion and SRPIN340 promoted transcription of the genes with a high number of exons (Fig. 2D, E). If these genes are not spliced correctly, cells would accumulate mRNAs with wrong open reading‐frames and premature stop‐codons, which should cause defects during translation.

We performed gene set enrichment analysis on our SLAM‐seq data to probe if CRPC cells mount an adaptive response to SRPK1 inhibition. This approach identified the ‘unfolded protein response’ (UPR) as the most significantly enriched gene set in response to both Endovion and SRPIN340 treatments (Fig. 2F; Fig. S2). Activation of the UPR likely represents an adaptive response to degrade the proteins made using the miss‐spliced mRNAs. In any case, UPR decreases protein synthesis and thereby decreases proliferation, whilst allowing recovery over time.

So far we have shown that Endovion causes similar effects on active transcription as does the well‐established SRPK1 inhibitor SRPIN340. Our data further shows that inhibition of SRPK1 selectively promotes transcription of the particularly long genes with a high number of exons. We propose that this stimulation effect is due to failure to recruit the splicing machinery on RNA Pol II, which allows more rapid mRNA‐production. If splicing of the genes is defective, we would expect accumulation of the misfolded proteins due to the faulty reading‐frames. Indeed, both Endovion and SRPIN340 activate the unfolded protein response. We hypothesize that prostate cancer cells with a particularly high levels of transcription would be susceptible to SRPK1 inhibition. Next, we wanted to identify a biomarker that could predict sensitivity against SRPK1 inhibition.

### 3.3 Inhibition of CDK12 promotes MYC signaling

**Fig. 3:**
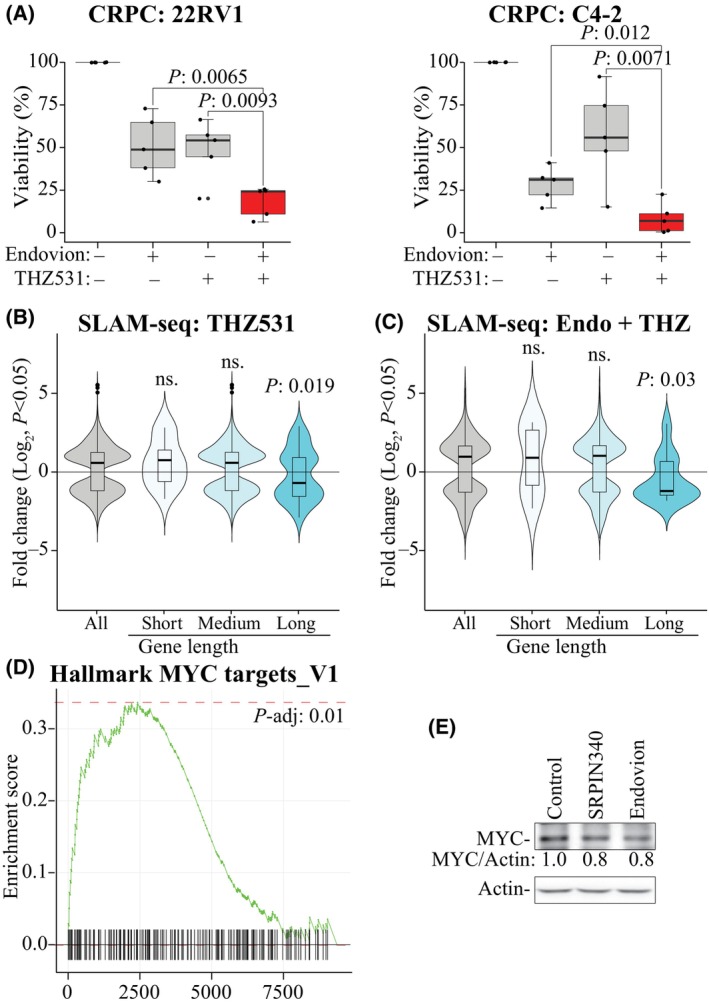
Endovion prevents cyclin‐dependent kinase 12 (CDK12)‐inhibition‐induced adaptive response. (A) Cell viability of CRPC cells after 4 days of treatment (n: 5 biological replicates, each three technical replicates). Endovion dose: 50 μM; THZ531 dose: 100 nM. Student's *t*‐test was used to confirm the statistical significance. (B, C) Analysis of the nascent transcription (SLAM‐seq) after 4 h treatment with 100 nM THZ531 or 100 nM THZ531 + 50 μM Endovion (22RV1 cells). Genes were grouped into three categories and significantly affected genes based on SLAM‐seq in each category are plotted. Significance was assessed using Student's *t*‐test, non‐significant: ns, n: 2. Number of genes in each category for THZ531, all: 2354, short: 15, medium: 2199, long: 140; and for Endovion + THZ531, all: 753, short: 8, medium: 710, long: 35. (D) Gene set enrichment analysis of the SLAM‐seq data (same as in 3C) identifies MYC targets as a gene set that is significantly activated in response to CDK12 inhibition in an SRSF protein kinase 1 (SRPK1)‐dependent manner (n: 2, see also Fig. S3B). Statistical test used: Kolmogorov–Smirnov test. (E) Inhibition of SRPK1 activity either with 20 μM SRPIN340 or 50 μM Endovion decreases MYC expression (22RV1 cell line and 24 h treatment). Three biological replicates.

We have shown that inactivation of *CDK12* is synthetically lethal with SRPK1 inhibition [10] and moved on to assess if inactivation of *CDK12* is a biomarker for sensitivity against Endovion. Endovion significantly sensitized two CRPC models to inhibition of CDK12 using THZ531 as determined using cell viability assays (Fig. 3A). We confirmed these effects using the colony‐formation experiments (Fig. S3A).

We hypothesized that combinatorial treatment with CDK12 and SRPK1 inhibitors would shut‐down the transcription, which thereby explains the observed anti‐proliferative effects. CDK12 is a transcription elongation kinase, which phosphorylates RNA Pol II to prevent premature transcription termination of particularly the long genes [5, 6]. When CDK12 is inhibited, transcription of the short genes is stimulated whilst transcription of the long is suppressed. Accordingly, inhibition of CDK12 stimulated transcription of the short genes, while the elongation of the long genes was severely impaired (Fig. 3B). Earlier, we noted that SRPK1 inhibition decreases transcription of the short genes but stimulates transcription of the long genes (Fig. 2C). We reasoned that combinatorial inhibition of SRPK1 and CDK12 would further suppress the overall transcription. In contrast, combining CDK12 inhibitor with Endovion stimulated transcription of the short genes and suppressed the long genes (Fig. 3C). Therefore the defects in RNA Pol II processivity are not the major reason explaining the lethality between combinatorial targeting of CDK12 and SRPK1.

At a loss for why the combination of CDK12 and SRPK1 inhibitors kills prostate cancer cells, we used gene set enrichment analysis. This approach identified MYC‐signaling as a significant gene set activated in response to CDK12 inhibition in an SRPK1‐dependent manner (Fig. 3D; Fig. S3B). When cells were treated simultaneously with inhibitors of CDK12 and SRPK1, MYC‐signaling was no longer significantly enriched (Fig. S3B). CDK12 inhibition stimulated transcription of the *MYC* gene as determined using SLAM‐seq and the treatment also stabilized the mRNA (Fig. S3C). Next, we confirmed that inhibition of SRPK1 with SRPIN340 and Endovion decreases the expression of MYC at the protein level, which explains at least partially why SRPK1 is important for the MYC‐driven transcriptional program (Fig. 3E; Fig. S3D). MYC functions as an amplifier of global transcription and it is the prototypical oncogene sufficient to transform a normal cell to cancerous [33]. In prostate cancer, overexpression of MYC confers the androgen‐independent phenotype, which leaves patients with no curative treatment options [34, 35].

**Fig. 4:**
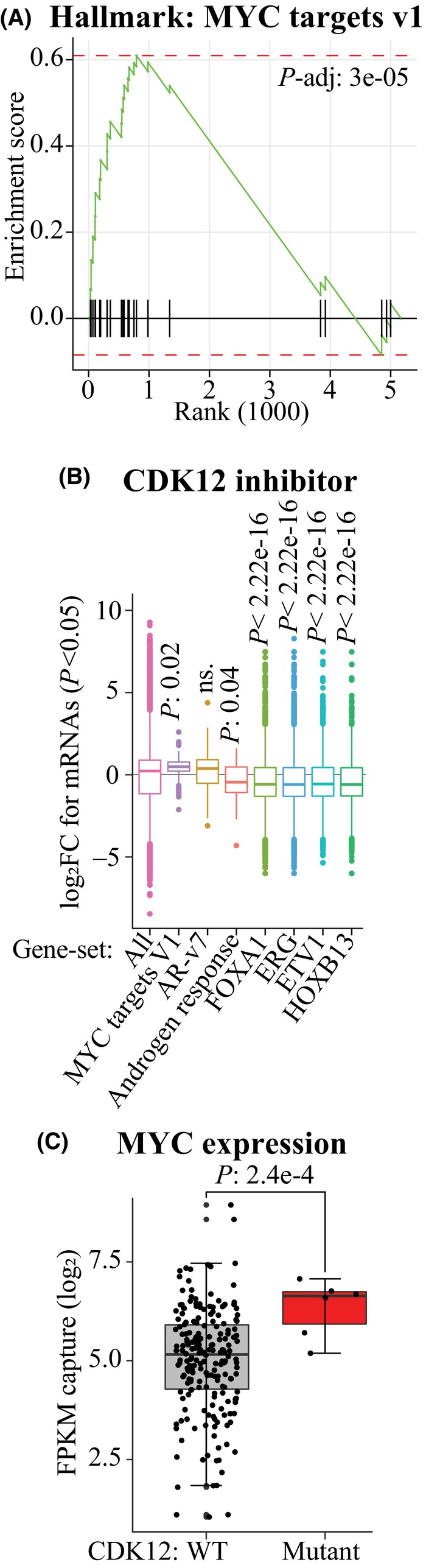
Decrease in CDK12 activity promotes MYC signaling *in vitro* and in patient samples. (A) Gene set enrichment analysis of LNCaP cells treated with 500 nM CDK12 inhibitor THZ531 for 6 h identifies MYC signaling as one of the most significantly affected gene sets (see also Fig. S3). This is a previously generated dataset that we downloaded from Genome Sequence Archive and re‐analyzed (HRA000724). (B) CDK12 inhibition promotes transcription of MYC and AR‐v7 target genes. We identified the genes belonging to each category from the GSEA database and by using published ChIP‐seq datasets (AR‐v7 [30], FOXA1 (GSE148926), ERG (GSM3223717), ETV1 (GSM1145322) and HOXB13 (GSE96652)). All the data presented in 4B were generated using the LNCaP cell line. Significance was assessed using Student's *t*‐test, non‐significant: ns. (C) *CDK12* mutant tumors overexpress MYC mRNA. We used the cBioPortal database to identify prostate cancer patients with truncating mutation in the *CDK12* gene from the SU2C/PCF Dream Team dataset and assessed the expression of the MYC mRNA in wild‐type (WT) and *CDK12*‐mutant tumors. Statistics are those reported in cBioPortal.

In order to gain further confidence into activation of MYC signaling in response to CDK12 inhibition in prostate cancer, we analyzed previously published RNA‐seq dataset from a different prostate cancer model, LNCaP. In support of our SLAM‐seq data, gene set enrichment analysis identified MYC signaling as one of the most enriched gene sets (Fig. 4A; Fig. S4).

It was unexpected that CDK12 inhibition would promote signaling through a specific transcription factor, because this kinase has been reported to be a positive regulator of all long genes [6]. To establish if the decrease in CDK12 activity promotes signaling through specific transcription factors, we relied on correlating our SLAM‐seq data with previously published ChIP‐seq datasets generated from the same cell line. We classified a gene as a target gene of a given transcription factor if there was a ChIP‐seq peak within the gene. Next, we compared how CDK12 inhibition affects transcription of all of the significantly affected genes to the target genes of the prostate cancer‐relevant transcription factors (MYC targets gene set, ligand‐independent androgen receptor variant AR‐v7, androgen response gene set, FOXA1, ERG, ETV1 and HOXB13). Strikingly, CDK12 inhibition selectively and significantly stimulated transcription of the MYC targets (Fig. 4B). In addition, we note that CDK12 inhibition also stimulated transcription of AR‐v7 target genes but this effect did not reach significance in our analysis. Previously, it has been established that CDK12 inactivation promotes signaling through AR‐v7 [36]. Here, we report for the first time that inhibition of CDK12 promotes the activity of the major oncogene MYC.

If inactivation of *CDK12* enhances MYC activity also in patient samples, this would explain why *CDK12* mutant prostate cancers are so aggressive. First, we assessed if the *CDK12* mutant tumors exhibit higher MYC expression. Indeed, MYC expression was significantly elevated in the tumors with *CDK12* truncating mutation when compared to the wild‐type tumors (Fig. 4C). Inactivation of *CDK12* is known to cause genomic amplifications [37, 38], and it is possible that the MYC‐locus and/or critical regulatory regions are amplified in the mutant tumors. However, establishing these go beyond the scope of the current manuscript.

To summarize our findings so far, decrease in CDK12 activity promotes MYC signaling in prostate cancer cells *in vitro* and in patient samples. Endovion does not augment CDK12 inhibitor‐induced effects on transcription elongation of the long genes, and defects in transcription elongation are therefore not the major reason explaining the lethality between combinatorial targeting of CDK12 and SRPK1. Instead, we show that Endovion prevents CDK12 inhibitor‐induced activation of MYC‐signaling by regulating MYC levels. However, we did not yet know if SRPK1 is important for the MYC‐driven proliferation of prostate cancer cells.

### 3.4 SRPK1 is required for the MYC‐driven proliferation of prostate cancer cells

**Fig. 5:**
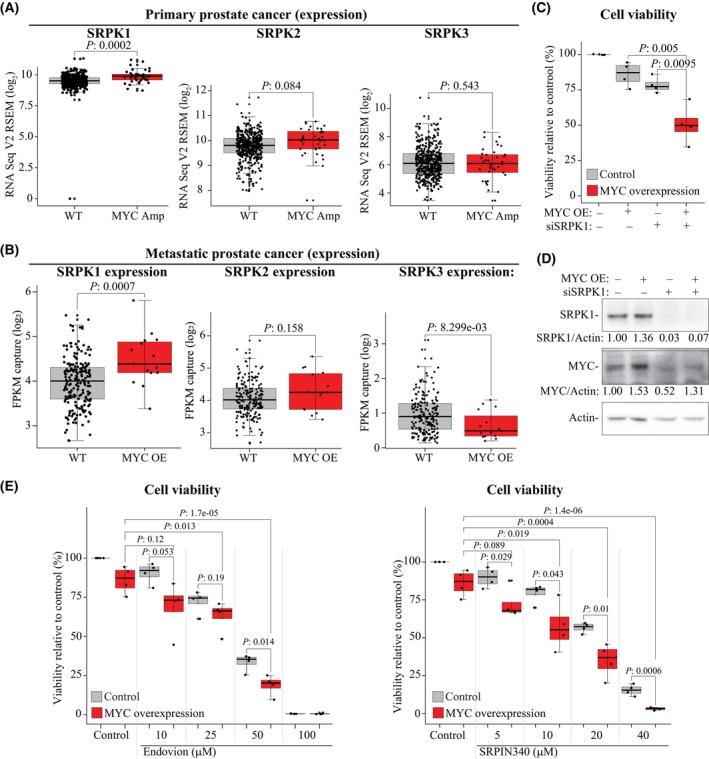
Overexpression of MYC sensitizes prostate cancer cells to SRSF protein kinase 1 (SRPK1) inhibition. (A) Primary prostate cancer tumors with *MYC* amplification overexpress SRPK1. We used the cBioPortal to identify prostate cancer patients of interest from the TCGA‐PRAD dataset and assessed the expression of the SRPK1‐3 mRNAs. Statistics are those reported in cBioPortal (WT, wild type: 454 and MYC AMP, MYC amplification: 37). (B) Metastatic prostate cancer tumors with increased MYC levels overexpress SRPK1. We used the cBioPortal to identify prostate cancer patients of interest from the SU2C/PCF Dream Team dataset and assessed the expression of the SRPK1‐3 mRNAs. Statistics are those reported in cBioPortal (WT: 193 and MYC OE, MYC overexpression: 15. (C) Overexpression of MYC sensitizes prostate cancer cells to SRPK1 knockdown. We used genetically engineered LNCaP‐MYC cell line, which enables overexpression of MYC by doxycycline addition. Data is from four biological replicates each having three technical replicates. Student's *t*‐test was used to confirm the statistical significance. (D) Confirmation of MYC overexpression by addition of 2 μG∙mL^−1^ doxycycline and knockdown of SRPK1 (LNCaP‐MYC cell line, representative of three biological replicates). (E) MYC overexpression sensitizes prostate cancer cells to SRPK1 inhibition using Endovion and SRPIN340 (LNCaP‐MYC cell line). MYC was induced for 24 h after which cells were treated as indicated for 4 days and the viability measured. Data is from four biological replicates each having three technical replicates. Student's *t*‐test was used to confirm the statistical significance.

We moved on to probe if SRPK1 is important for the MYC‐driven proliferation of prostate cancer cells. First, we identified prostate cancer patients with increased MYC activity by selecting primary prostate cancer patients with *MYC* amplification and metastatic prostate cancer patients with increased MYC expression. Indeed, increased MYC activity was associated with a significant overexpression of SRPK1 but not with increased expression of SRPK2 or SRPK3, the other two SRPK‐genes (Fig. 5A,B). Accordingly, we note a significant positive correlation between expression of MYC and SRPK1 (Fig. S5).

We used LNCaP‐MYC prostate cancer model system in which MYC expression can be activated by addition of doxycycline [19] to probe if the increased MYC activity sensitizes prostate cancer cells to SRPK1 inhibition. First, we performed SRPK1 knockdown, which was toxic to prostate cancer cells overexpressing MYC (Fig. 5C,D; Fig. S6). In addition, overexpression of MYC significantly sensitized prostate cancer cells to SRPK1 inhibition using SRPIN340 and Endovion (Fig. 5E). These data establish inactivation of *CDK12* as a biomarker for acquired sensitivity to SRPK1 inhibition.

Endovion represents a candidate drug to be assessed in clinical trial against prostate cancers with *CDK12* inactivation. Such a trial would require a multi‐institute collaboration because inactivation of *CDK12* is relatively rare even in CRPC. Loss of *CDK12* increases the immunogenicity of the tumor [3, 37, 39], and it may be possible to further boost this by inhibiting SRPK1. In designing clinical trial assessing Endovion against prostate cancer, it is important to remember that whilst the compound has been deemed safe in healthy individuals [18], weakening of the overall physic in patients with the late‐stage CRPC may result in unexpected toxicities that limit the dose that can be safely achieved. We propose that *CDK12* inactivation is a biomarker of sensitivity against SRPK1 inhibition using Endovion.

## 4 Discussion

In this project, we set out to establish if Endovion induces similar transcriptional effects as does the SRPK1 inhibitor SRPIN340, and to identify a biomarker that would predict sensitivity against Endovion. We show that Endovion dose‐dependently inhibits proliferation of CRPC cells (Fig. 1B). Unexpectedly, both Endovion and SRPIN340 stimulated transcription of the genes that have a high exon count (Fig. 2D,E). This was unexpected, because splicing is required for generation of the productive mRNAs. In this context, it is important to remember that we measured the actual mRNA synthesis after only 4 h treatment time and not the absolute mRNA levels; it is therefore possible that the overall transcription decreases over a longer period of time. We hypothesize that when SRPK1 is inhibited, splicing is decoupled from transcription, and the actual RNA synthesis is more efficient. In agreement with this, it has previously been established that RNA Pol II speed affects recruitment of the splicing machinery [40, 41]. In the future, it is important to establish the crosstalk between RNA Pol II phosphorylation, recruitment of the splicing machinery and the RNA Pol II speed.

Previously, it has been established that Endovion additionally targets drug efflux pumps [18], and some of the anti‐proliferative effects are likely explained through these targets. Importantly, however, our data show that Endovion induces highly similar effects on the nascent transcriptome as does the well‐established SRPK1 inhibitor SRPIN340. In the follow‐up studies, it is of a great interest to probe how Endovion affects splicing and compare these effects to other SRPK1 inhibitors. This can be achieved using tools such as rMATS, SUPPA, and MISO, which accept standard RNA‐seq data [42]. As Endovion is the only clinically assessed SRPK1 inhibitor, it also represents an exciting compound for further development in translating SRPK1 inhibitors to patient‐care.

Inactivation of the transcription elongation kinase *CDK12* sensitizes CRPC cells to SRPK1 inhibition [10]. We confirmed that depletion of CDK12 activity sensitizes CRPC cells to SRPK1 inhibition also using Endovion (Fig. 3A). Using gene set enrichment analysis, we discovered that inhibition of CDK12 activity promotes MYC signaling in an SRPK1‐dependent manner (Fig. 3D; Fig. S3B). It was unexpected that inactivation of *CDK12*, a kinase that is considered as a general regulator of transcription elongation [43], would selectively promote signaling through a specific transcription factor. We therefore confirmed these effects using another prostate cancer model, and observed that CDK12 inhibition significantly promotes MYC‐signaling (Fig. 4A). CDK12 inhibitor‐induced transcription of genes was selective to MYC and AR‐v7 because inhibition of CDK12 downregulated the mRNAs regulated by the other prostate cancer‐relevant transcription factors including FOXA1, ERG, ETV1, and HOXB13 (Fig. 4B). We confirmed the clinical relevance of these findings by showing that the decrease in CDK12 activity is associated with increased MYC expression in prostate cancer patient samples (Fig. 4C) and we also revealed a positive correlation between MYC and SRPK1 expression (Fig. S5).

In the future, it is important to establish how *CDK12* inactivation actually promotes MYC‐signaling. This likely occurs through a multitude of mechanisms, but there are two obvious supported by the literature and our findings. First, decrease in CDK12 activity promotes transcription of the short genes [5, 6], and MYC itself is a fairly short gene (coding sequence 1362 and 1365 nucleotides for different isoforms) [28]. Accordingly, CDK12 inhibition stimulated MYC transcription in CRPC cells (Fig. S3C). Second, inactivation of *CDK12* can cause *MYC* amplification and/or *MYC* enhancer amplification through faulty DNA repair. Mechanistically, it is well‐established that inactivation of *CDK12* causes amplifications across the genome [37, 38] and the highly transcribing regions are more susceptible to DNA damage [44, 45]. We therefore propose that the *MYC*‐locus is more susceptible to DNA damage and therefore faulty DNA repair in cells where CDK12 activity is compromised. Understanding these effects will enable rational drug design, particularly in the context of prostate cancer but in addition potentially in other cancers with *CDK12* inactivation. Overall, our data demonstrate that the loss of *CDK12* promotes MYC signaling in an SRPK1‐dependent manner, which predicts that cells overexpressing MYC should be sensitive to SRPK1 inhibition as well.

We used prostate cancer patient samples and *in vitro* model to demonstrate that the increase in *MYC* dosage and expression causes dependency on SRPK1. Prostate cancer patients with amplification and/or higher expression of MYC in their tumors also overexpressed SRPK1 but not the other SRPK‐genes (Fig. 5A). Next, we used prostate cancer model system in which MYC can be overexpressed. This system revealed that MYC overexpression sensitizes prostate cancer cells to SRPK1 inhibition (Fig. 5C,E). In brief, *CDK12* inactivation and MYC overexpression render cells dependent on the otherwise non‐essential kinase SRPK1.

Overexpression of MYC has been shown to sensitize cells to core components of the spliceosome [46]. This exciting discovery may have limited benefit to patients, because the compounds targeting the core components of the spliceosome have caused severe toxicity in clinical trials [8, 9]. In the future, it is important to probe in more detail if MYC overexpression in the absence of *CDK12* inactivation sensitizes cells to SRPK1 inhibition, as this could open an attractive strategy to target the MYC‐driven cancers. MYC is notoriously challenging to directly inhibit [47], which increases the value of our discoveries. In essence, targeting the non‐essential splicing regulator SRPK1 may offer a selective treatment strategy against the MYC‐driven cancers.

Genetic depletion of SRPK1 is embryonically lethal [48], which raises concerns in developing SRPK1‐targeting therapies. However, and importantly, SRPK1 is a non‐essential gene in most cell types [10], and here we identify a biomarker of sensitivity against Endovion, inactivation of the *CDK12* gene. When planning the future clinical trials assessing Endovion against the late state and pre‐treated prostate cancer patients, it is important to remember that whilst the compound has been deemed safe in healthy individuals [18], weakening of the overall status of the heavily treated patients may result in unexpected toxicities that limit the dose that can be safely achieved. In aggregate, these data support further development of Endovion, and also more specific inhibitors of SRPK1, as a therapeutic strategy against tumors with defects in transcription elongation.

## 5 Conclusions

Inactivation of *CDK12* characterizes an aggressive sub‐group of prostate cancers but it is not known why loss of this transcription elongation kinase promotes proliferation of the affected cells. Here, we show that the loss of CDK12 promotes MYC activity in both patient tumors and cell line models. Finally, we show that CDK12‐driven MYC signaling depends on SRPK1 and we identify an SRPK1 inhibitor Endovion as a compound that targets the affected cells.

## Conflict of interest

The authors have no conflicts of interest to declare.

## Author contributions

JL performed most of the experiments, analyzed the majority of the data and contributed to manuscript writing. AG and HMI collected SLAM‐seq samples and AG also evaluated combination treatments with Endovion and CDK12 inhibitor (CTG). HMI conceptualized the project, supervised the study, obtained resources and wrote the manuscript. All authors have contributed to developing the project and writing the manuscript.

### Peer review

The peer review history for this article is available at https://www.webofscience.com/api/gateway/wos/peer‐review/10.1002/1878‐0261.13666.

## Supporting information


**Fig. S1.** Knockdown of SRPK1 sensitizes prostate cancer cells to Endovion.
**Fig. S2.** Unfolded protein response is the most significantly affected gene set in response to SRPK1 inhibition.
**Fig. S3.** Endovion prevents MYC‐driven adaptive signaling induced by inhibition of CDK12 using THZ531.
**Fig. S4.** Gene set enrichment analysis identifies MYC signaling as one the most enriched gene sets after CDK12 inhibition.
**Fig. S5.** The expression of MYC and SRPK1 is positively correlated in prostate cancer patient samples and cell lines.
**Fig. S6.** Densitometry analysis of the western blot data presented in main Fig. 5D.

## Data Availability

Sequencing data has been deposited to GEO database under the accession number GSE236089. The article is associated with six Supplementary figures provided as a single pdf document.
