# Supplementary material for: CDK12‐inactivation‐induced MYC signaling causes dependency on the splicing kinase SRPK1
Source: Mol Oncol. 2024 May 22;18(10):2510–23. doi: 10.1002/1878-0261.13666 (PMC11459032; doi:10.1002/1878-0261.13666)
Supplement: Supplementary file 1 — Fig. S1. Knockdown of SRPK1 sensitizes prostate cancer cells to Endovion. Fig. S2. Unfolded protein response is the most significantly affected gene set in response to SRPK1 inhibition. Fig. S3. Endovion prevents MYC‐driven adaptive signaling induced by inhibition of CDK12 using THZ531. Fig. S4. Gene set enrichment analysis identifies MYC signaling as one the most enriched gene sets after CDK12 inhibition. Fig. S5. The expression of MYC and SRPK1 is positively correlated in prostate cancer patient samples and cell lines. Fig. S6. Densitometry analysis of the western blot data presented in main Fig. 5D. [file MOL2-18-2510-s001.pdf]

Supporting information: Supplementary figures and figure legends

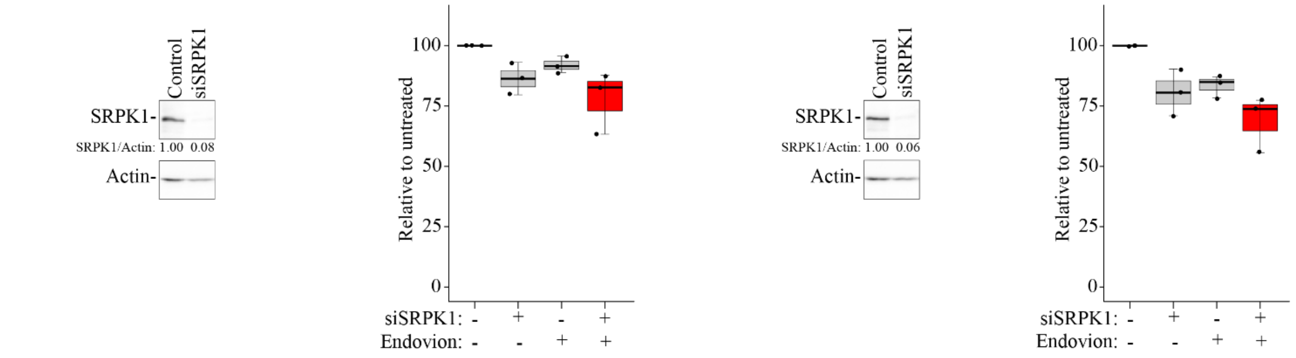

Supplementary figure 1. Knockdown of SRPK1 sensitizes prostate cancer cells to Endovion. Knockdown of SRPK1 was confirmed using western blotting after three days. For cell viability-assays, knockdown was performed for 24 hours, cells treated with 25 µM Endovion and viability measured using CellTiterGlo-assay. Data shown is from three biological replicates each having three technical replicates.

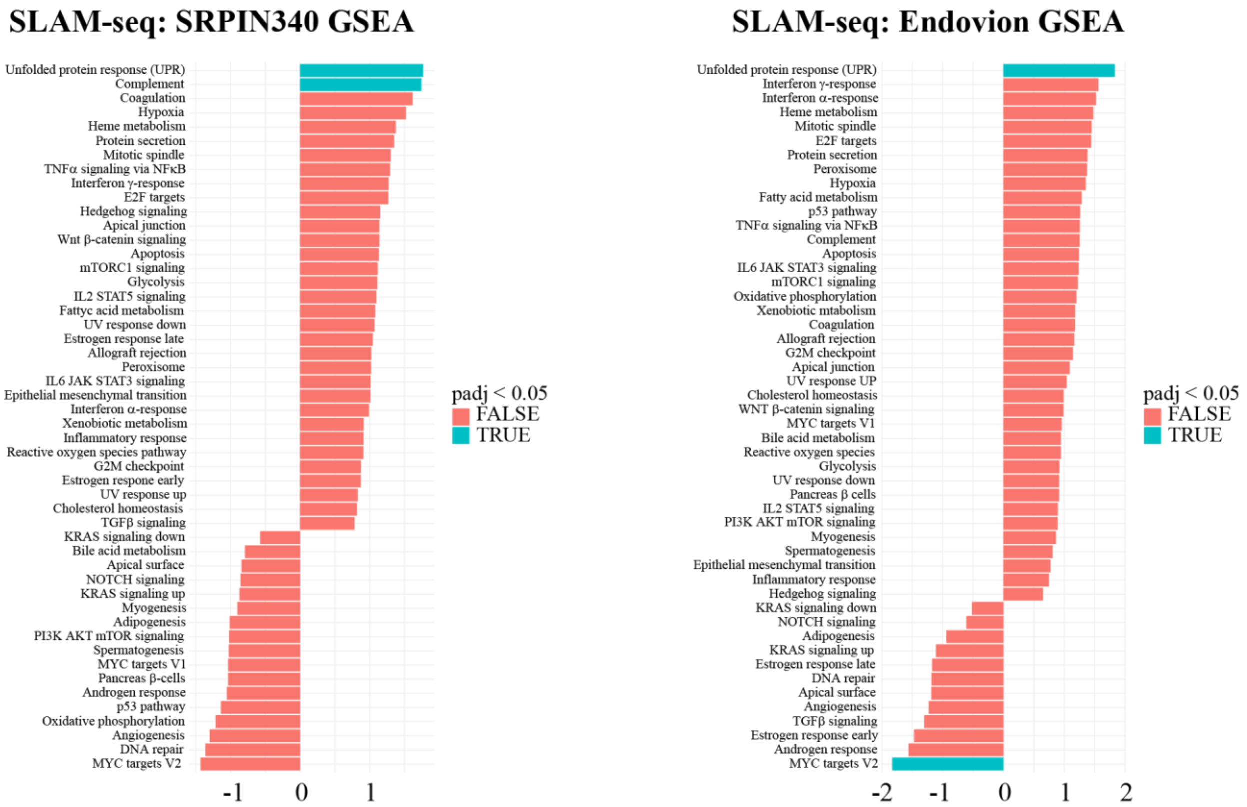

Supplementary figure 2. Unfolded protein response is the most significantly affected gene set in response to SRPK1 inhibition. Differences in active transcription after 4 hours treatment with 20 µM SRPIN340 or 50 µM Endovion were identified using SLAM-seq. Gene set enrichment analysis of the SLAM-seq data was used to identify the most significantly affected gene sets. SLAM-seq data was generated using 22RV1 cell line.

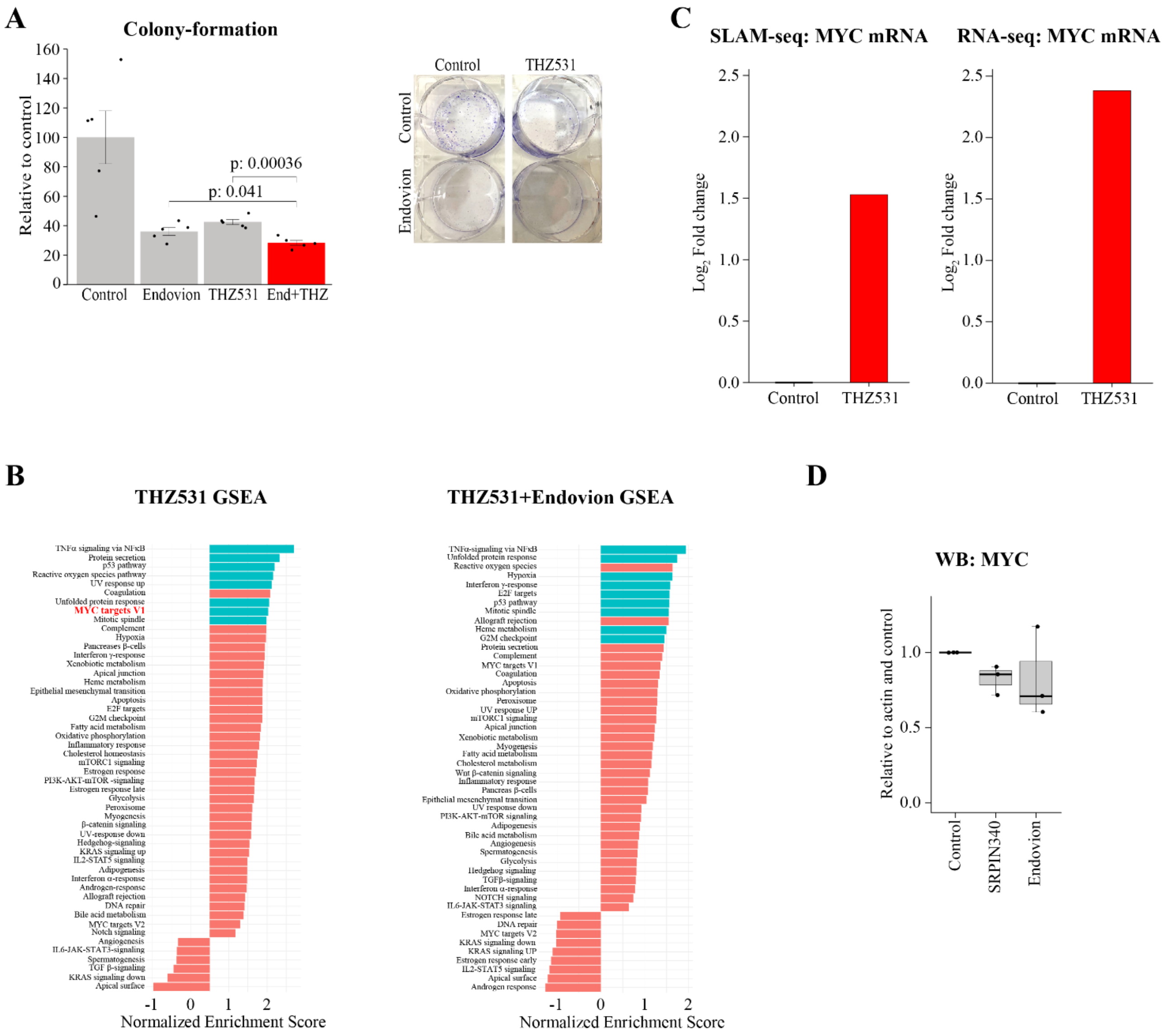

Supplementary figure 3. Endovion prevents MYC-driven adaptive signaling induced by inhibition of CDK12 using THZ531. A) Colony-formation of 22RV1 cells in one week after the indicated treatments (Endovion: 40 µM and THZ531: 40 nM). Data shown is from five biological replicates and student's t-test was used to confirm the statistical significance. **B)** Differences in active transcription after 4 hours treatment with 100 nM THZ31 alone or in combination with 50 µM Endovion was identified using SLAM-seq (22RV1 cell line). Gene set enrichment analysis of the SLAM-seq data was used to identify the most significantly affected gene sets. **C)** Log_2 fold change of the MYC mRNA as determined using SLAM-DUNK and RNA-seq ($p<0.01$ as determined using DESeq2). **D)** Densitometry analysis of MYC and actin proteins to confirm that 20 µM SRPIN340 and 50 µM Endovion decrease MYC levels. Cells were treated for 24 hours and the data presented is from three biological replicates.

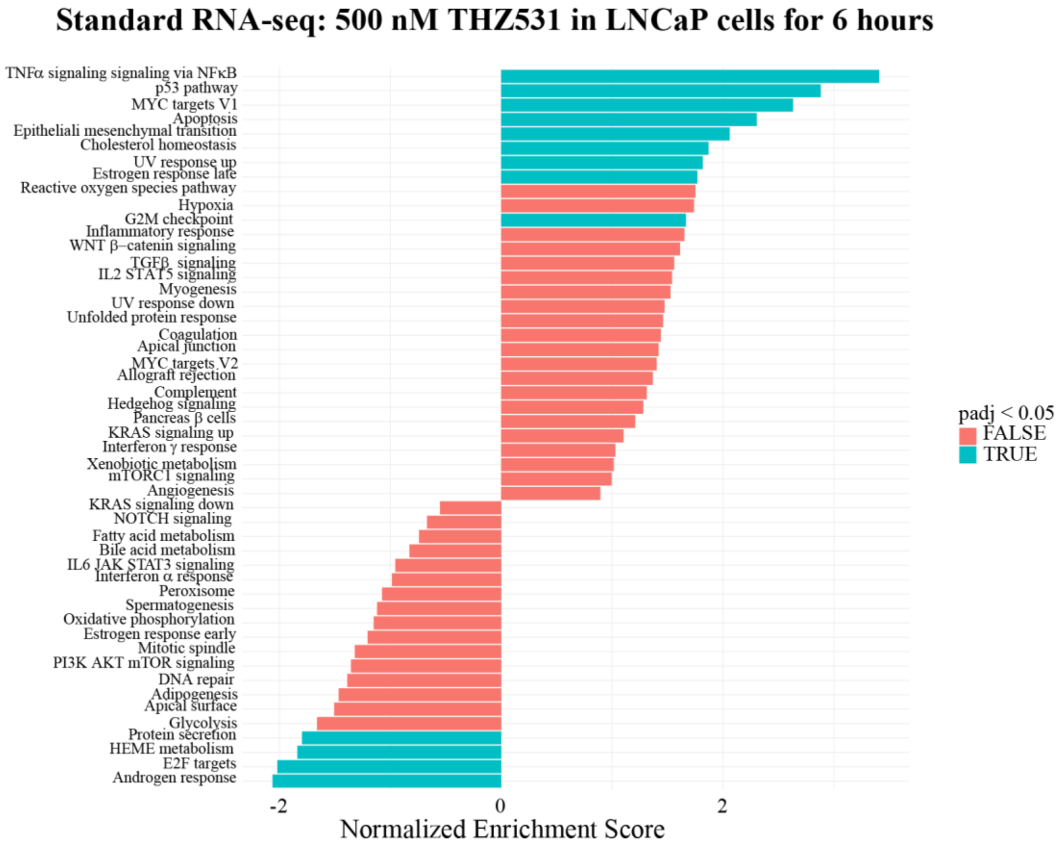

Supplementary figure 4. Gene set enrichment analysis identifies MYC signaling as one the most enriched gene sets after CDK12 inhibition. Gene set enrichment analysis of previously published RNA-seq data (HRA000724). In brief, authors treated LNCaP cells for 6 hours with 500 nM THZ531, purified RNA and performed RNA-seq. We re-analyzed these data and performed gene set enrichment analysis.

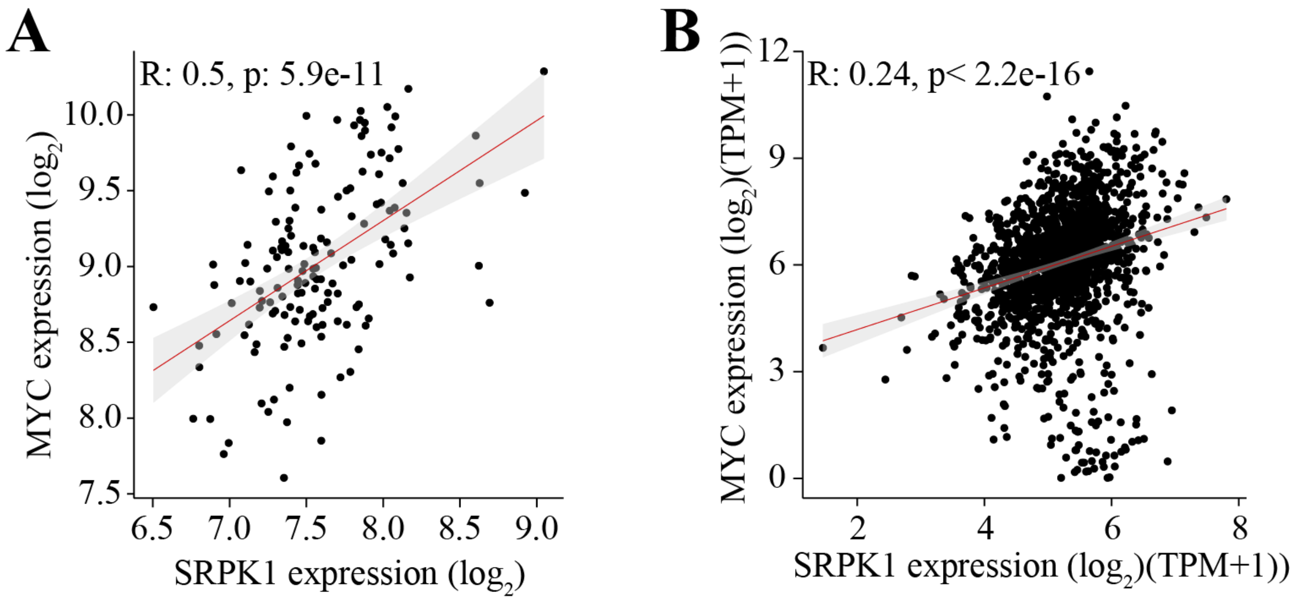

Supplementary figure 5. The expression of MYC and SRPK1 is positively correlated in prostate cancer patient samples and cell lines. A) The expression of MYC and SRPK1 in prostate cancer patient samples (data was accessed through the Betastasis-tool, using the Taylor *& al.* dataset [1]). **B)** The expression of MYC and SRPK1 in different cell line models (n: 1475) using the data accessible through the DepMap-portal (https://depmap.org/portal/). To assure the correlation, we also used the Bayes factor-analysis. For the patient data-set, Bayes factor was 177640743 and for the cell line dataset, the value was 2.6e+17 (extreme evidence to support that the expression of MYC and SRPK1 is positively correlated).

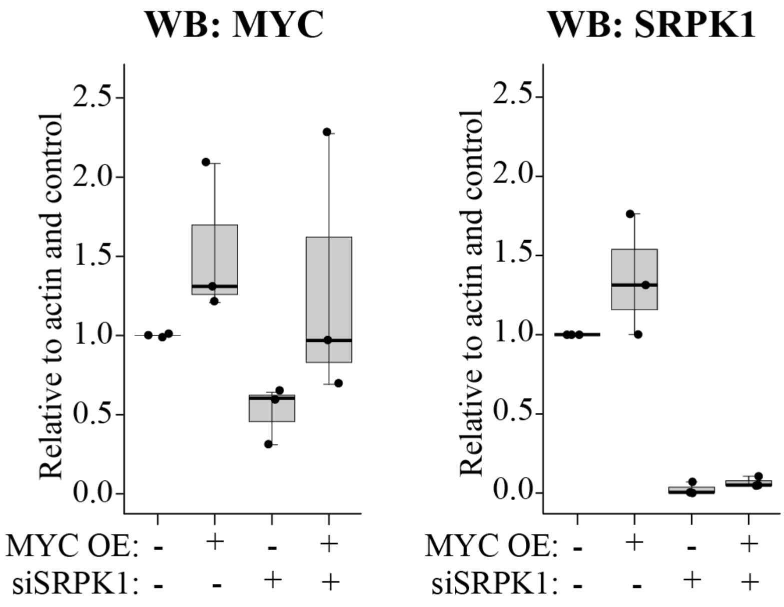

Supplementary figure 6. Densitometry analysis of the western blot data presented in main figure 5D. Confirmation of MYC overexpression by addition of 2 µG/mL doxycycline and knockdown of SRPK1 (three biological replicates).

References of the Supplementary files:

1 Taylor BS, Schultz N, Hieronymus H, Gopalan A, Xiao Y, Carver BS *et al*. Integrative genomic profiling of human prostate cancer. *Cancer Cell* 2010; 18: 11-22.
